# Characterization of an Equine α-S_2_-Casein Variant Due to a 1.3 kb Deletion Spanning Two Coding Exons

**DOI:** 10.1371/journal.pone.0139700

**Published:** 2015-10-07

**Authors:** Julia Brinkmann, Tomas Koudelka, Julia K. Keppler, Andreas Tholey, Karin Schwarz, Georg Thaller, Jens Tetens

**Affiliations:** 1 Institute of Animal Breeding and Husbandry, Christian-Albrechts-Universität Kiel, Kiel, Germany; 2 Systematic Proteomics & Bioanalytics, Institute for Experimental Medicine, Christian-Albrechts-Universität Kiel, Kiel, Germany; 3 Institute of Human Nutrition and Food Science, Division of Food Technology, Christian-Albrechts-Universität Kiel, Kiel, Germany; Montana State University, UNITED STATES

## Abstract

The production and consumption of mare’s milk in Europe has gained importance, mainly based on positive health effects and a lower allergenic potential as compared to cows’ milk. The allergenicity of milk is to a certain extent affected by different genetic variants. In classical dairy species, much research has been conducted into the genetic variability of milk proteins, but the knowledge in horses is scarce. Here, we characterize two major forms of equine α_S2_-casein arising from genomic 1.3 kb in-frame deletion involving two coding exons, one of which represents an equid specific duplication. Findings at the DNA-level have been verified by cDNA sequencing from horse milk of mares with different genotypes. At the protein-level, we were able to show by SDS-page and in-gel digestion with subsequent LC-MS analysis that both proteins are actually expressed. The comparison with published sequences of other equids revealed that the deletion has probably occurred before the ancestor of present-day asses and zebras diverged from the horse lineage.

## Introduction

Horses are of minor importance in global dairy production, but mare’s milk has traditionally been consumed in Mongolia, Kazakhstan, Kyrgyzstan or Tajikistan [1]. The global amount of production is not exactly known, but it has been estimated that approximately 30 million people worldwide are regularly consuming mare’s milk [2]. Also in Europe, especially in Italy, Hungary, The Netherlands and Germany, the production and consumption of mare’s milk have gained more and more importance; roughly 1 million kg of mare’s milk are produced in Europe [3]. This increased interest is mainly based on positive health effects. The milk of horses and donkeys is e.g. tolerated by the majority of children suffering from cow’s milk protein allergy, a condition that affects approximately 2% of infants when nourished with milk replacements on cow milk basis [4,5]. Moreover, positive effects of mare’s milk consumption on diseases like atopic dermatitis [6], Morbus Crohn [7] or cardiovascular diseases [8] have been reported.

The composition of equine milk, and especially the milk protein fraction, is very different from that of cows’ milk. It is lower in fat and protein, but has a high lactose content similar to what is found in human milk [9,1]. While in cattle the casein fraction accounts for the major part of the total milk protein, the casein to whey ration in horses is around 1.1:1, which more closely resembles human milk [9,1]. In fact, it has been reported that the balance between caseins and whey proteins is a major determinant of cow’s milk allergenicity [10] possibly giving an explanation for the low allergenic potential of horse milk. However, there is also strong evidence that genetic milk protein variants affect the allergenicity of milk protein based on the presence or absence of particular epitopes [11,12]. While there has been intense research into the genetic variability of milk proteins in ruminants and especially in dairy cows [13], the knowledge about equine milk protein variation is scarce, especially for the caseins. However, in the donkey different variants of α_S2_-casein have been described, also involving a large deletion exons 4–6 [14].

In the present study, we characterized a major protein variant arising from a 1.3 kb in-frame-deletion covering two exons and proved the protein by means of LC-MS based analytics at the protein level.

## Material and Methods

### Animals and Samples

Genomic DNA was extracted from hair samples of 193 domestic horses from 8 different breeds that are actually used for mare’s milk production in Germany applying a modified Miller protocol[15]. The animals were selected to be as unrelated as possible. Hair samples were obtained from 14 different private studs with permission of and in cooperation with the owners by pulling out several hairs from the mane or the tail. Furthermore, individual milk samples were collected from four Haflinger mares with known genotype. These samples were taken by the owners during routine milking of the mares. In concordance with German Animal welfare legislation, these sampling procedures do not require a permission or approvement.

### DNA Sequencing

Primer pairs were designed to amplify the coding exons contributing of equine *CSN1S2* and adjacent intronic regions using the Primer 3 software [16] based on the genomic reference sequence of the casein gene *CSN1S2* (Acc. No NC_009146.2). A further Primer pair (Forward: 5’- GGAAAAGATTTGTGAGCCATTTG–3’, Reverse: 5’- GCTGGATAATTGCTCAACACTCA–3’) was designed to specifically amplify the entire region of *CSN1S2* encompassing the deletion. PCR amplification and DNA sequencing were done as previously described [17]. The obtained sequences were analyzed and compared with the genomic reference sequence (Acc. No. NC_009168.2) using the software Sequencher 4.9 (Gene Codes Corp., Ann Arbor, MI).

### RNA Isolation from Milk Samples and cDNA Synthesis

Individual milk samples were obtained from four mares with known deletion genotype. An aliquot of 40 ml was centrifuged at 6,000 g for 10 minutes. The supernatant including the milk fat layer was discarded and remaining milk fat was thoroughly removed with alcohol wipes. The cell pellet was washed three times with 1x phosphate buffered saline. Cells were homogenized using QIAShredder columns (Qiagen, Hilden, Germany) and total RNA was isolated using the Qiagen RNeasyMini Kit (Qiagen, Hilden, Germany) according to the manufacturer’s instructions. The isolated RNA was transcribed into cDNA using SuperScript® III First-Strand Synthesis SuperMix kit (Invitrogen) with oligo-dT primers. PCR amplification and sequencing was done with primers located in the untranslated regions (Forward: 5’-TGCCTGCACTTTCTTGTCTTCCA–3’, Reverse: 5’-TGCACAGTCTTCATTTGGCTTGA–3’).

### Protein and Peptide Analysis

Individual milk samples of two mares with known genotype were used for protein analysis. These samples were dialyzed to remove lactose, subsequently freeze dried and stored at -18°C for 4 months. Lyophilized milk powder was dissolved in Laemmli buffer (1x) at a concentration of 2 mg/mL and 10 and 20 μg of crude protein was loaded onto a 12% SDS-PAGE gel (150V for 85 min). Gel bands were destained, reduced and alkylated and then subsequently in gel-digested overnight with trypsin (60 ng) using standard protocols. Peptides were extracted from the gel, dried down using vacuum centrifugation and resuspended in 3% acetonitrile (ACN) and 0.1% trifluroacetic acid (TFA) before being analyzed by LC-MS.

Nano-UHPLC-MS was performed on an UltiMate 3000 RSL Nano/Cap System (Thermo Fisher Scientific, Bremen, Germany) coupled online to an Orbitrap QExactive (Thermo Fisher Scientific). Samples were desalted for 4 minutes (Acclaim PepMap100 C–18, 300 μm I.D. x 5 mm, 5 μm, 100 Å, Thermo Fisher Scientific) at a flow rate of 30 μL/min using 3% ACN, 0.1% TFA. An Acclaim PepMap100 C–18 column (75 μm I.D. x 500 mm, 2 μm, 100 Å, Thermo Fisher Scientific) was used for analytical separation at a flow rate of 300 nL/min using binary gradients of buffers A (0.05% FA) and B (80% ACN and 0.04% FA). The elution used gradient steps of 5–50% B (4–30 min) and 50–90% B (30–35 min) followed by an isocratic wash (90% B, 35–45 min) and column re-equilibration (5% B, 45–60 min) steps.

MS scans were acquired in the mass range of 300 to 2,000 m/z at a resolution of 70,000. The ten most intense signals were subjected to HCD fragmentation using a dynamic exclusion of 15 s. MS/MS parameters—minimum signal intensity: 1000, isolation width: 3.0 Da, charge state: ≥2, HCD resolution: 15,000, Normalized collision energy of 25. Lock mass (445.120025) was used for data acquired in MS mode.

HCD spectra were searched using Proteome Discoverer 1.4 (1.4.0.288, Thermo Fisher Scientific) with the Sequest-HT search algorithm against the complete reviewed and unreviewed *Equus caballus* database (28,188 sequences, downloaded 2015.07.16) with common contaminants (ftp://ftp.thegpm.org/fasta/cRAP/) appended. The following database search settings were used: MS tolerance; ± 10 ppm, MS2 Tolerance; 0.02 Da, enzyme specificity; trypsin with up to three missed cleavages allowed. Carbamidomethylation on cysteine residues was set as a fixed modification while, oxidation on methionine, and phosphorylation on serine and threonine residues was set as a variable modification. Only peptides which were identified with medium confidence (FDR <5%) were included.The mass spectrometry proteomics data have been deposited to the ProteomeXchange Consortium (http://www.proteomexchange.org) via the PRIDE partner repository with the dataset identifier PXD002834.

## Results and Discussion

### DNA Sequencing and Mutation Screening

**Fig 1:**
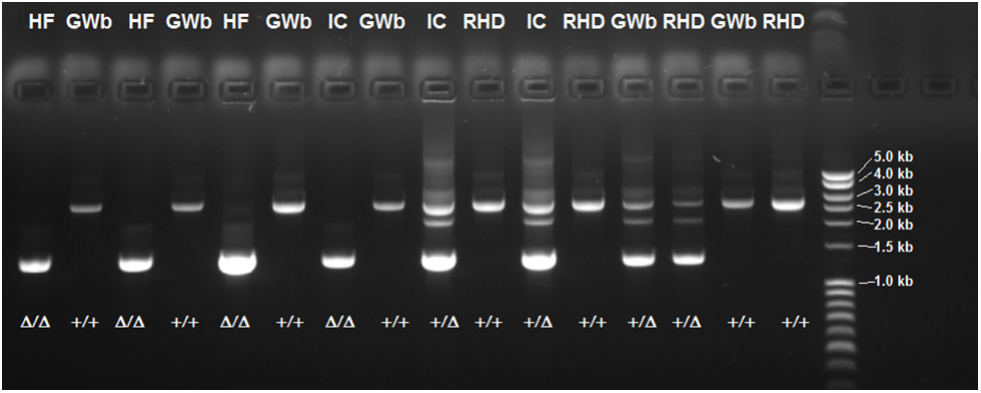
Agarose gel electrophoresis of PCR products spanning the 1.3 kb deletion. The upper visible band corresponds to the long variant (denoted as +), the lower one to the short variant containing the deletion (denoted as Δ). Only in heterozygotes, a third band with a size of approximately 2.1 kb is visible, which is possibly arising from asymmetric hybridization of the alleles due to the presence of a duplication. The breeds of the corresponding samples are given above the lanes (RHD = Russian Heavy Draft, GWb = German Warmblood, IC = Icelandic horse, HF = Haflinger).

**Fig 2:**
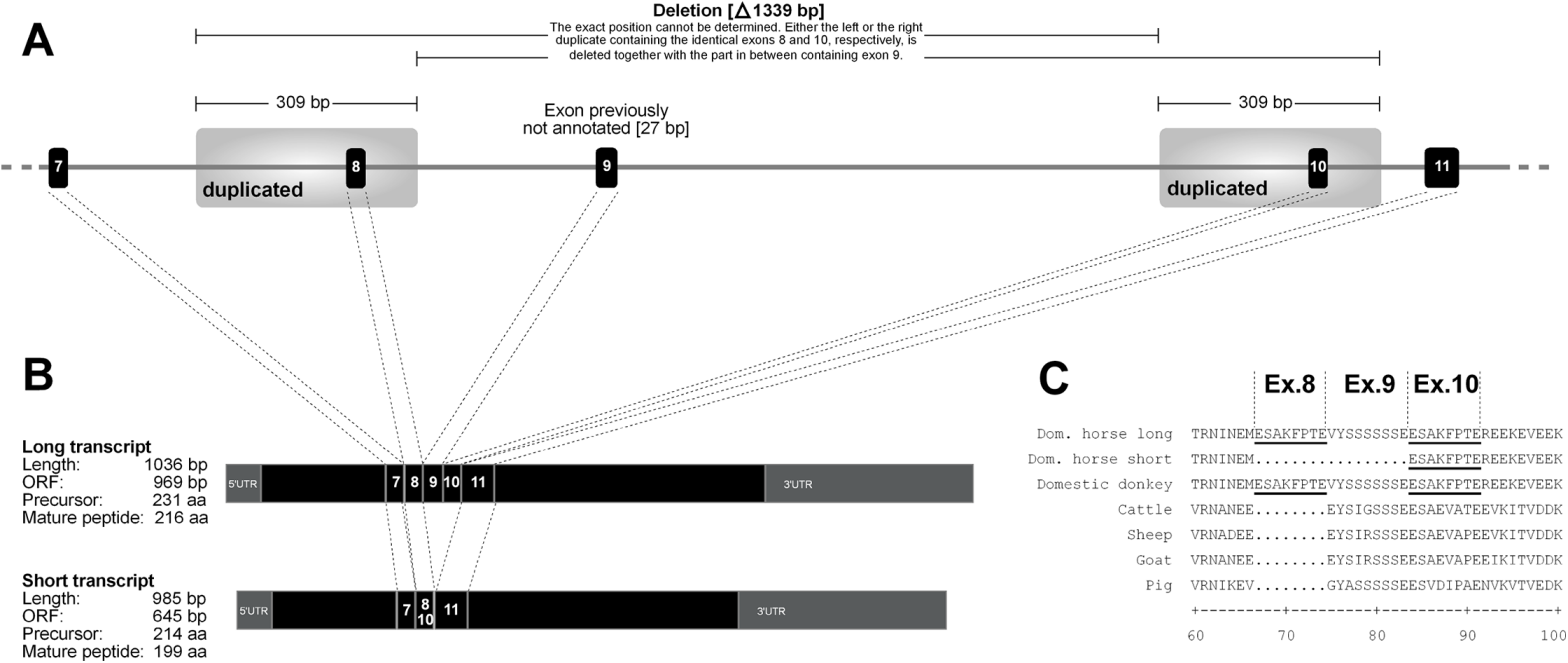
Structure of the long and short equine α_s2_-casein variants. A. Genomic organization of the respective gene segment. Grey shading indicates the equid specific 309 bp duplication comprising coding exons 8 and 10, respectively. The 1.3 kb in-frame-deletion is indicated above the figure. B. Structures the resulting transcript variants. C. Protein alignment of available ungulate α_s2_-casein protein sequences.

The current annotation of the equine *CSN1S2* gene (GeneID 100327035) is based on the mRNA reference sequence NM_001170767.2 containing 15 coding exons with an open reading frame of 645 bp. In an attempt to resequence the open reading frame using exon flanking primer pairs, we recognized that the PCR reactions for exons 8 and 9 consistently failed in particular horses. In order to unravel the possible cause for this phenomenon, we amplified a 2.6 kb fragment spanning the entire region. While the expected product was obtained from samples that had been successfully amplified before, the product obtained from initially unsuccessful samples was found to be approximately 1.3 kb shorter (Fig 1). Subsequent Sanger sequencing of the products revealed the presence of a 1,339 bp deletion in the short variant (Fig 2A), while the long product was found to completely correspond to the genomic reference sequence (NC_009146.2). Analysis of this sequence revealed the presence of a 309 bp duplication of the region encompassing exon 8 of the gene (Fig 2A). Because this duplication is located exactly at the boundary of the deletion, the exact position cannot be determined, i.e. it cannot be ruled out, whether the upstream or downstream duplicate is involved in the deletion.

A total of 193 horses belonging to 8 breeds (Table 1) were tested for the presence of the deletion by PCR and subsequent agarose gel electrophoresis (Fig 1). The deletion was found to be present in all analyzed breeds; the highest frequencies of 0.36 and 0.25 were observed in Haflinger and Icelandic horses, respectively. Notably, these breeds are common in mare’s milk production, especially the Haflinger breed is widely used. This might possibly indicate an effect of the mutation or a certain casein-haplotype on milk yield as this is e.g. the case in cattle [18,19].

**Table 1:** Frequencies of the 1.3 kb deletion in different horse breeds.

Breed	n	ins/ins^a^	ins/del^a^	del/del^a^	Frequency of deletion
Crossbred^b^	21	14	6	1	0.19
Criollo	27	24	3	0	0.06
Fjord Horse	3	2	1	0	-^c^
Haflinger Horse	39	17	16	6	0.36
Icelandic Horse	24	13	10	1	0.25
Quarter Horse	20	16	3	1	0.13
Russian Heavy Draft	24	20	3	1	0.10
German Warmblood	35	33	2	0	0.03
TOTAL	193	139	44	10	0.17

^a^ ins = long variant corresponding to the genomic reference NC_009146.2; del = short variant encompassing the 1,339 bp deletion spanning two coding exons.

^b^ A synthetic cross involving German Riding Pony, Haflinger Horse, Connemara Pony and New Forrest Pony; bred for milk yield.

^c^ The frequency in Fjord horses is not reported with respect to the small sample size, but the breed is included in the total values.

### Analysis of Transcripts

**Fig 3:**
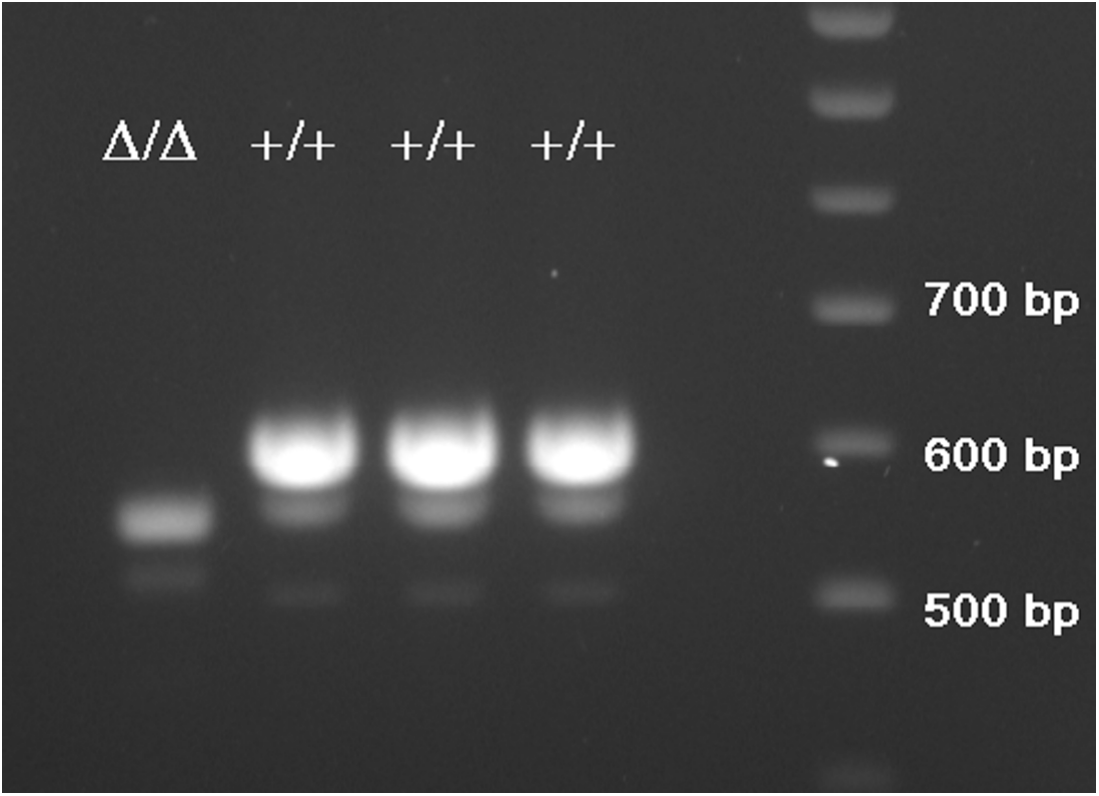
Agarose gel electrophoresis of equine *CSN1S2* cDNA. The RNA was isolated from the milk of a mare being homozygous for the deletion (Δ/Δ).and three mares homozygous for the long variant (+/+).

The duplicated region within the 1.3 kb deletion contains a coding exon with a length of 24 bp. The two copies were found to be completely identical including intact splice sites. However, only one of the identical exons is present in the current RefSeq transcript NM_001170767.2. Thus, it was unclear which exons are transcribed and whether both variants are transcribed at all. Therefore, we purified total RNA from the skimmed milk of four mares, three of them being homozygote for the long and one for the short variant, respectively. After reverse transcription, the *CSN1S2* transcripts were amplified using primers located in the untranslated regions. Agarose gel electrophoresis of the PCR products revealed a difference of approximately 50 bp between the alternatively homozygote animals (Fig 3) showing that both, a long and a short transcript, were actually expressed. Subsequently, the open reading frames of both transcripts were sequenced. The difference was found to be due to a 51 bp in-frame insertion/deletion after exon eight encompassing the duplicated exon as well as a previously not annotated exon that perfectly aligns within the 1.3 kb deletion (Fig 2A and 2B). Exon numbering was consequently adapted counting the newly annotated exon as exon 9 and the duplicate of exon 8 as exon 10. Although the genome assembly (EqCab2.0) comprises the long variant, the RefSeq transcript NM_001170767.2 used for annotation represents the short variant. A BLAST search showed that both transcripts had been reported before (GenBank KP658381.1 and KP658382.1) and both variant transcripts have recently been added to the unreviewed UniProt database (Acc. No. A0A0C5DH76 and D2KAS0), but no further information or publication is available. The transcript sequences from the current study are available under the accession numbers KT368778 and KT368779.

### Comparative Analysis

Translations of the long and short transcript, respectively, were aligned to available protein sequences of domestic donkey (Acc. No. CAV00691.1 [20]), cattle (Acc. No. NP776953.1), sheep (Acc. No. NP_001009363.1), goat (Acc. No. NP_001272514.1) and pig (Acc. No. NP_001004030.1). From Fig 2C it can be seen that the duplication of exon 8/10 is unique to donkey and horse, while the equine exon 9 appears to have a homologous sequence in other ungulates. A BLAST search against the genome assembly of Przewalski’s horse (Burgud assembly, CGF0000696695.1), a species that separated from the ancestral population of domesticated horses 38–72 kyr BP ago [21,22], also revealed the presence of the long variant including the duplicated exon. Furthermore, the small read archive data sets of a Middle Pleistocene horse, the “Thistle Creek horse” sequenced by Orlando and colleagues [22] (BioProject Accession PRJNA205517), as well as of several asses and zebras sequenced by Jónsson and colleagues ([23], BioProject Accession PRJEB7446) were checked for reads either falling into the deleted region (indicating the long variant) or being split at the boundaries (indicating the short variant). Only a single read almost perfectly aligning within the deletion was found in the Middle Pleistocene horse, which might point to the presence of the long variant. In the Somalian wild ass (*E*. *asinus somalicus*), we only identified the long variant, while both alleles were found in the Tibetian Kiang (*E*. *kiang*). The sequenced Onager (*E*. *hemionus onager*) was found to be homozygous for the deletion. These findings indicate that the duplication event giving rise to an additional coding exon as well the deletion might be specific to equids and must both have occurred before the ancestor of present-day asses and zebras dispersed into the Old World 2.1–3.4 Mya [23]. However, all analyzed zebras [23] (Hartmanns Mountain zebra, *E*. *zebra hartmannae*; Grevy zebra, *E*. *grevyi*; Böhm’s plains zebra, *E*. *quagga boehmi* as well as the extinct Quagga, *E*. *q*. *quagga*) were found to be homozygous for the complete deletion. It seems also possible that the deletion initially occurred in horses and represents the result of a gene flow between horses and ass species. It has been shown that this has played a significant role in equid evolution [23].

### Protein and Peptide Analysis

**Fig 4:**
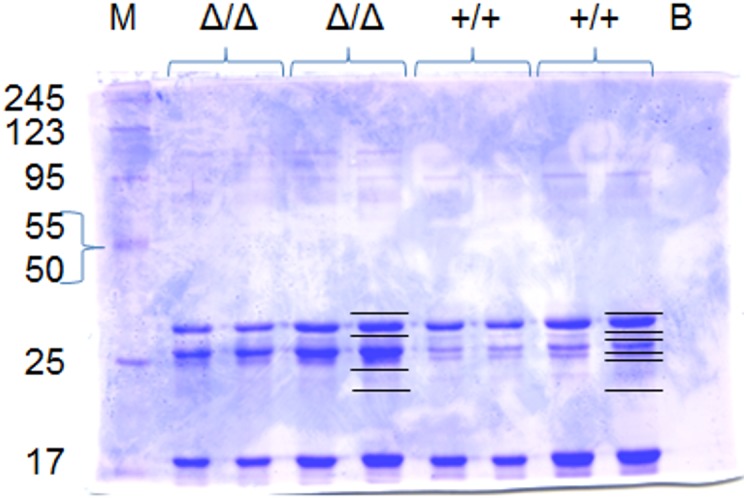
SDS-PAGE of crude mare’s milk being homozygous for the deletion (Δ/Δ) and homozygous for the long variant (+/+). Ten and 20 μg of crude milk from both mares was loaded in duplicate. Excised bands that were in-gel digested with trypsin and later analyzed by LC-MS are indicated. M (Roti^®^-Mark Bicolor protein standard); B (Blank, Laemmli buffer).

**Fig 5:**
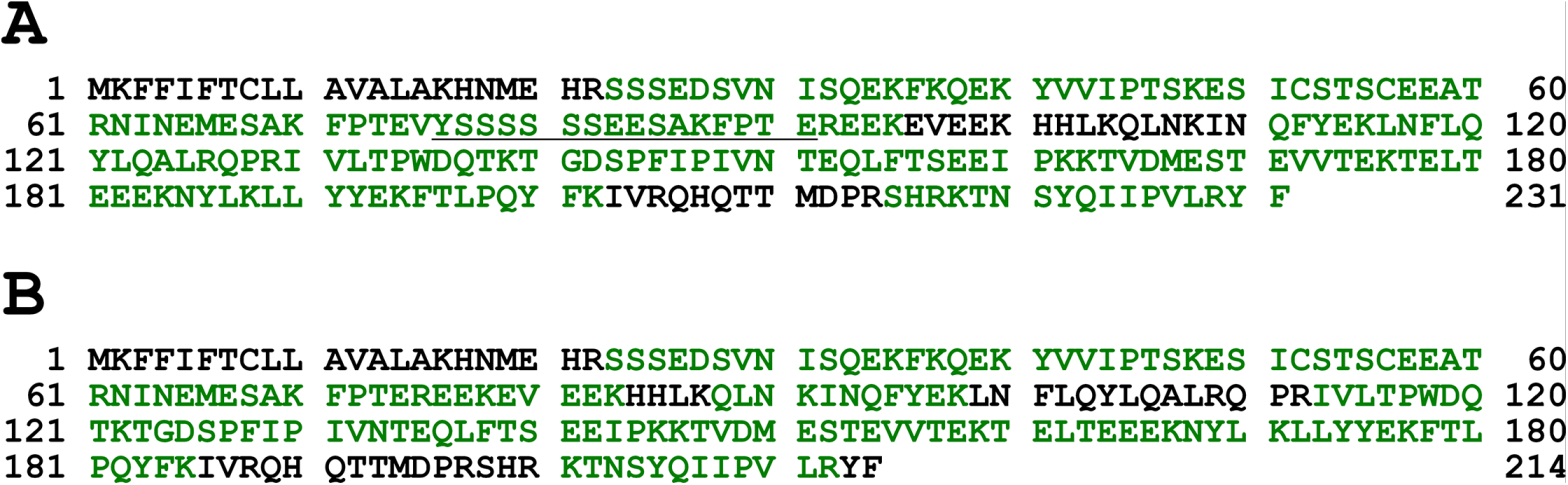
Combined sequence coverage (all bands excised) of α_S2_-casein in mare with +/+ genotype (A) and Δ/Δ genotype (B). From the +/+ genotype only unique peptides were identified for the longer α_S2_-casein form (Accession no. A0A0C5DH76, 231 AAs). Sequence which is unique to A0A0C5DH76 is underlined. For the Δ/Δ genotype only unique peptides were identified for the shorter alphaS2-casein form (Accession no. D2KAS0, 214 AAs). Sections in green represents parts of the protein which were identified by the sequest-HT algorithm.

Lyophilized milk powder from two mares being homozygote for the long and short variant, respectively, were analyzed using SDS-PAGE resulting in different patterns of distinct bands (Fig 4). In-gel trypsin digestion and analysis by LC-MS revealed the presence of unique peptides only for the long form of α_s2_-casein (A0A0C5DH76) in milk of the animal homozygous for the insertion, i.e., peptides FPTEVYSSSSSSEESAK, FPTEVYSSSSSSEESAKFPTER, FPTEVYSSSSSSEESAKFPTEREEK and NINEMESAKFPTEVYSSSSSSEESAK. Interestingly, these peptides were identified in both phosphorylated (singly phosphorylated at different residues) and non-phosphorylated forms. Evidence for multiple phosphorylations on these peptides was also observed. Unique peptides for the short form of α_s2_-casein (D2KAS0) were only identified in milk from the mare homozygous for the deletion, i.e., NINEMESAKFPTER, NINEMESAKFPTEREEK, NINEMESAKFPTEREEKEVEEK (Fig 5, Table 2). As commonly observed in MS based protein analytics, a 100% sequence coverage was not reached; however, the proteotypic peptides identified allowed clearly to distinguish the two equine α_S2_-casein variants. Therefore, it can be concluded that both protein variants differing in length by 17 aa are expressed. The comparative analysis has shown that the long variant is probably the equid specific ancestral variant, but the deletion also seems to have been present before zebras and asses diverged from horses. Thus, we propose to term the long variant CSN1S2*A and the short variant CSN1S2*B.

**Table 2:** Bands excised from a mare with +/+-genotype (upper part) and Δ/Δ genotype (lower part), respectively, and major milk proteins identified are shown (only those with > 20 PSMs). AlphaS2-casein with accession no. A0A0C5DH76 is the long form (231 AA) while alphaS2-casein with accession no. D2KAS0 is the shorter form of alphaS2-casein (214 AAs). Unique peptides were only identified for the long form of alphaS2-casein (A0A0C5DH76) in the +/+-mare, while unique peptides for the short form of alhphaS2-casein (D2KAS0) were identified only in milk from the Δ/Δ-mare.

Band	Accession	Description	Coverage (%)	#Unique Peptides	#Peptides	#PSMs
**Mare homozygous for long variant (+/+)**						
1	Q9GKK3	ß-casein	35.68	9	9	87
	D2KAS0	α_s2_-casein	49.53	0	13	35
	A0A0C5DH76	CSN1S2 protein	45.89	0	13	35
2	Q9GKK3	ß-casein	32.78	8	8	45
	A0A0C5DH76	α_s2_-casein	72.73	2	17	45
	D2KAS0	α_s2_-casein	64.02	0	15	43
	P82187	κ-casein	23.78	7	9	40
	Q95KZ7	α_s1_-casein	41.35	1	12	31
3	Q9GKK3	ß-casein	35.68	11	11	138
	Q95KZ7	α_s1_-casein	45.67	2	15	47
	A0A0C5DH76	α_s2_-casein	58.01	1	14	31
	D2KAS0	α_s2_-casein	54.67	0	13	30
	P82187	κ-casein	23.78	4	6	21
4	Q95KZ7	α_s1_-casein	45.67	2	15	67
	Q9GKK3	ß-casein	35.68	10	10	35
	A0A0C5DH76	α_s2_-casein	60.17	2	16	31
	D2KAS0	α_s2_-casein	54.67	0	14	29
5	A0A0A1E470	Immunoglobulin lambda light chain variable region (fragment)	46.46	2	12	62
	A0A0C5DH76	α_s2_-casein	71.86	3	20	59
	D2KAS0	α_s2_-casein	63.08	0	17	52
**Mare homozygous for short variant (Δ/Δ)**						
1	Q9GKK3	ß-casein	35.68	10	10	80
2	Q9GKK3	ß-casein	35.68	12	12	139
	Q95KZ7	α_s1_-casein	45.67	2	16	75
	Q8SPR1	α_s1_-casein	63.68	0	18	65
	D2KAS0	α_s2_-casein	73.36	2	20	60
	A0A0C5DH76	α_s2_-casein	58.44	0	18	58
	P82187	κ-casein	29.73	3	6	34
3	D2KAS0	α_s2_-casein	73.36	1	24	82
	Q9GKK3	ß-casein	35.68	11	11	80
	A0A0C5DH76	α_s2_-casein	67.97	0	23	78
	Q95KZ7	α_s1_-casein	31.25	2	9	24

Generally, the milk proteome is very complex both due to the presence of genetic variants and posttranslational modifications [24]. Recent proteomic studies [25,26] have demonstrated considerable microheterogeneity for equine caseins, especially κ-casein [25]. However, these studies did not report any findings regarding α_S2_-casein, probably due to its very low concentration in horse milk [1]. However, Ochirkhuyag et al. [27]reported the presence of two distinct bands for this protein. In our study, both genetic variants as well as differently phosphorylated peptides have been detected for α_S2_-casein.

## Conclusion

Within the current study, we have characterized two major variants of equine α_S2_-casein, which we named CSN1S2*A and CSN1S2*B. The variation is due to a 1.3 kb in-frame deletion involving two coding exons corresponding to 17 amino acid residues. One of those exons has arisen from a duplication that is probably specific to the equid lineage. We verified both genomic variants at the transcript as well as the protein level and were able to demonstrate that these variants are also segregating in asses, meaning that they are likely to have occurred before the first ancestor of present-day asses and zebras dispersed into the Old World 2.1–3.4 Mya.
